# Temperature-Responsive Molecular Assemblies Using Oligo(Ethylene Glycol)-Attached Polyamidoamine Dendron Lipids and their Functions as Drug Carriers

**DOI:** 10.3390/jfb11010016

**Published:** 2020-03-13

**Authors:** Takuya Hashimoto, Yuji Hirai, Eiji Yuba, Atsushi Harada, Kenji Kono

**Affiliations:** Department of Applied Chemistry, Graduate School of Engineering, Osaka Prefecture University, 1-1 Gakuen-cho, Naka-ku, Sakai, Osaka 599-8531, Japan; sv108049@edu.osakafu-u.ac.jp (T.H.); 35615u@ube-ind.co.jp (Y.H.); biopolymer@chem.osakafu-u.ac.jp (K.K.)

**Keywords:** temperature-responsive nanocarrier, polyamidoamine dendron lipid, morphological change, doxorubicin

## Abstract

Temperature-responsive nanocarrier systems using external stimuli are one of the most widely investigated stimuli-responsive strategies because heat is easy and safe to use for hyperthermia and controlled drug delivery. Polyamidoamine dendron lipids (PAMAM-DLs) composed of PAMAM dendron as head group and two alkyl chains can exhibit temperature-responsive morphological change through the attachment of suitable moieties to terminal of PAMAM dendron. In this study, oligo(ethylene glycol)s including ethoxy- or methoxy-diethylene glycols were attached to the terminals of PAMAM-DL, and temperature-responsive properties of their self-assemblies were evaluated by calorimetric and turbidity measurements. In the evaluation of temperature-responsive properties, ethoxy diethylene glycol (EDEG)-attached PAMAM-DL composed of two saturated alkyl chains and PAMAM dendron with 1st generation had lipid bilayer structure and suitable cloud point for the application as drug carrier. In vitro performances of the assemblies combining EDEG-attached PAMAM-DLs with cholesteryl-oxy-poly(ethylene glycol) (PEG-Chol) was evaluated using doxorubicin (DOX) as an anticancer drug. Cellular uptake of DOX-loaded EDEG-attached PAMAM-DL/PEG-Chol assemblies was promoted at 42 °C rather than 37 °C, resulting in an effective decrease in cell viability.

## 1. Introduction

Recent advancement in nanocarrier-based drug delivery system has contributed to therapeutic strategies. In the design of nanocarriers, stimuli-responsivity is important for controlled drug delivery. Stimuli are classified into endogenous stimuli such as pH decrease, high glutathione concentration and matrix metalloproteinases and external stimuli such as light, ultrasound and magnetic field [1,2,3,4,5]. In particular, temperature-responsive nanocarrier system using external stimuli are one of the most widely investigated stimuli-responsive strategies because heat is easy and safe to use for hyperthermia and controlled drug delivery [6]. As temperature-responsive nanocarrier system, the molecular assemblies including micelles and vesicles composed of amphiphilic polymers and molecules are widely investigated, and it was reported that temperature-responsive liposomes showed effective therapeutic effect for the local release of drugs by combining with hyperthermia [4,7].

We have studied the self-assembly of polyamidoamine dendron lipids (PAMAM-DLs) composed of PAMAM dendron as head group and two alkyl chains as hydrophobic tail from the both fundamental viewpoints and the application as nanocarriers [8,9,10]. The introduction of temperature-responsive moieties including alkyl amide groups which are the side groups of temperature-responsive polymers such as poly(*N*-alkyl acrylamide) and poly(*N*-vinyl alkyl amide) can provide PAMAM dendrimer and PAMAM-DLs self-assembly exhibiting temperature-responsive morphological change [11,12,13]. When isobutyramide (IBAM) groups were introduced to the periphery of PAMAM-DL, the morphology of IBAM-attached PAMAM-DL self-assembly changed from spherical vesicle to fibrous structure with hexagonally packed cylinders by increasing temperature above cloud point [8,13]. In addition, the cloud points of PAMAM-DL self-assembly could be controlled by introducing IBAM and acetamide groups with varying ratios to PAMAM-DL. Such temperature-responsive properties of PAMAM-DL self-assembly can expect that temperature-specific release of anticancer drug from the assemblies with the morphological change by heating. However colloidal stability under physiological conditions as well as biocompatibility are required for the application of temperature-responsive PAMAM-DL assembly as a temperature-responsive nanocarrier.

**Figure 1:**
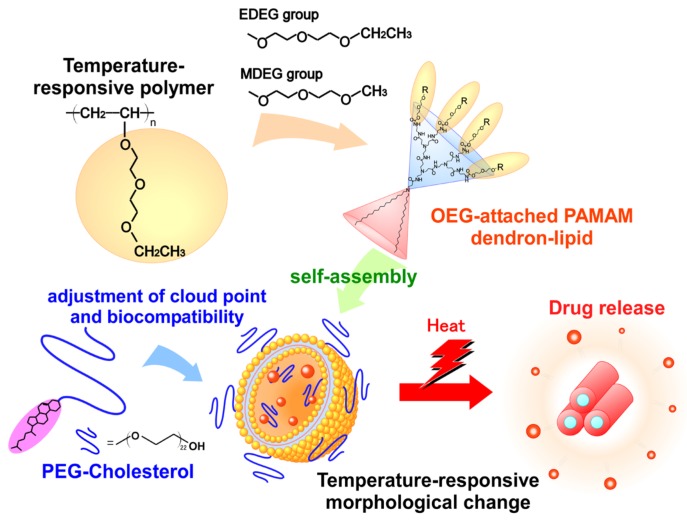
Schematic image of OEG-attached PAMAM dendron-lipid assembly as temperature-responsive nanocarrier.

Here we focused on oligoethylene glycol (OEG) units as temperature-responsive moieties. It has been reported that the introduction of OEG units to the polymers or metal nanoparticles provides temperature-responsivity, biocompatibility and the inhibition of nonspecific protein adsorption [14,15,16]. Indeed, we confirmed that the introduction of OEG units to PAMAM dendrimers and hyperbranched polyglycidols provided temperature-responsivity [17,18]. In this study, OEG-attached PAMAM-DLs were synthesized and temperature-responsive properties of their self-assemblies were evaluated by calorimetric and turbidity measurements. For the application as a temperature-responsive nanocarrier, cholesteryl-oxy-poly(ethylene glycol) (PEG-Chol) was combined to temperature-responsive PAMAM-DL self-assembly for the adjustment of cloud point and improvement of colloidal stability and biocompatibility (Figure 1). Furthermore, the availability of OEG-attached PAMAM-DL/PEG-Chol assemblies was confirmed using doxorubicin (DOX), an anticancer drug, through the evaluation of in vitro performances including cellular uptake, intracellular distribution and anticancer effect of DOX-loaded assemblies.

## 2. Results and Discussion

**Figure 2:**
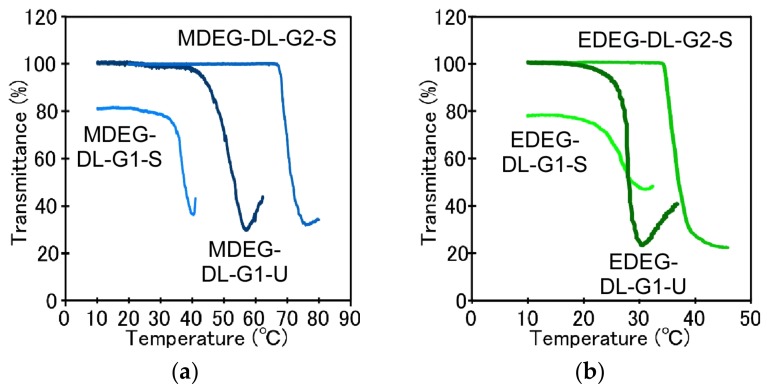
Change in transmittance of OEG-attached PAMAM-DLs dispersions with an increase in temperature. (**a**) MDEG-attached PAMAM-DLs, (**b**) EDEG-attached PAMAM-DLs. OEG-attached PAMAM-DLs were suspended in 10 mM phosphate and 140 mM NaCl at pH 7.4.

Temperature-responsive behavior of OEG-attached PAMAM-DLs dispersions (MDEG-DL-G1-S, EDEG-DL-G1-S, MDEG-DL-G2-S, EDEG-DL-G2-S, MDEG-DL-G1-U and EDEG-DL-G1-U) was evaluated by monitoring the change in transmittance at 500 nm with an increase in temperature (Figure 2). Remarkable difference in transmittance at 10 °C was observed. Although MDEG-DL-G2-S, EDEG-DL-G2-S, MDEG-DL-G1-U and EDEG-DL-G1-U dispersions were transparent solution (≈100%T), MDEG-DL-G1-S and EDEG-DL-G1-S dispersions had relatively low transmittance (ca. 80 %T). To compare the size distributions of OEG-attached PAMAM-DLs self-assembly, DLS measurements were performed at 10 °C. As expected from relatively low transmittance at 10 °C, OEG-attached PAMAM-DLs bearing saturated hydrocarbon chains and the dendron with 1st generation (MDEG-DL-G1-S and EDEG-DL-G1-S) formed large aggregates. On the other hand, another OEG-attached PAMAM-DLs could form the self-assemblies with diameter of several tens nm. When increasing temperature, steep decrease in transmittance was observed for all dispersions, indicating temperature-responsive aggregation (Figure 2). The change in average diameter in DLS with an increase in temperature were agreed with the change in transmittance (Figure S1), and average diameter steeply increased at cloud points of OEG-attached PAMAM-DLs. Such temperature-responsive behavior of OEG-attached PAMAM-DLs might be induced by the dehydration with the accumulation of OEG units introduced at the terminal groups of the dendron on the surface of the self-assembly. We have already reported the importance of accumulation of suitable moieties like OEG and *N*-isopropylamide etc. in the expression of temperature-responsive property by using PAMAM dendrimer with various generations. Cloud points which exhibit steep decrease in %T were summarized in Table 1.

**Figure 3:**
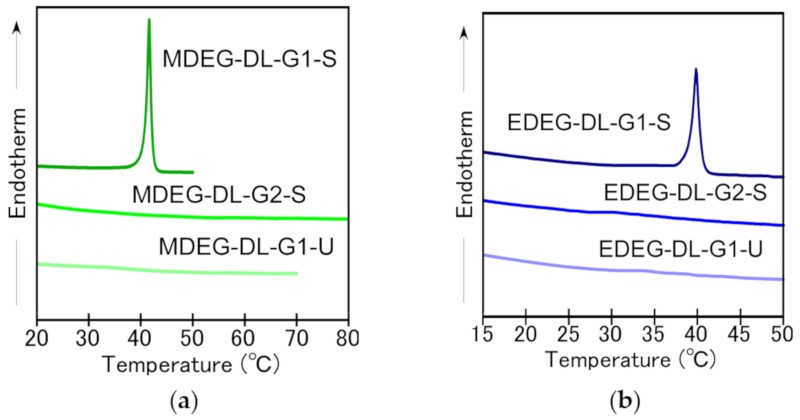
Microcalorimetric endotherms of OEG-attached PAMAM-DLs suspended in 10 mM phosphate and 140 mM NaCl at pH 7.4. (**a**) MDEG-attached PAMAM-DLs, (**b**) EDEG-attached PAMAM-DLs.

Cloud points of OEG-attached PAMAM-DLs decreased as the terminal group became more hydrophobic from methoxy to ethoxy and the cloud point increased as the generation of dendron increased from first to second generation. Li et al. evaluated the effect of OEG moieties to cloud points of OEG-attached dendrimer and dendronized polymers [19,20]. They confirmed that the change of terminal groups from methoxy into ethoxy showed strong influence on cloud points, in which the polymers bearing ethoxy terminal of the OEG moiety showed low cloud point compared with the case of methoxy terminal. We have also reported similar effects of terminal groups on OEG-attached hyperbranched polyglycidols [17]. Increase in cloud point with an increase in dendron generation might be due to hydrophilicity of interior of PAMAM dendron including tertiary amines and amides. The effect of hydrophilicity of dendron interior was evaluated through pH-dependence of cloud points of EDEG-DL-G2-S (Figure S2). Although cloud points were kept constant (35 °C) at pH 7 and higher, the decrease in pH from neutral to acidic provided an increase in cloud point. Increase of cloud point with a decrease in pH is induced by an increase in hydrophilicity and a decrease in the density of OEG moieties at the surface with protonation of tertiary amine in the dendron interior. Additionally, cloud points are influenced by hydrocarbon chains of OEG-attached PAMAM-DLs. Because it is known that the saturation of hydrocarbon chains of lipids influences to their membrane characters including fluidity, molecular packing. OEG-attached PAMAM-DLs bearing unsaturated hydrocarbon chains showed lower cloud points compared with OEG-attached PAMAM-DLs bearing saturated hydrocarbon chains. This might be due to the difference in the fluidity of lipid bilayer. DSC measurements were performed for OEG-attached PAMAM dendron-lipids self-assembly (Figure 3). Endothermic peaks were detected for MDEG-DL-G1-S and EDEG-DL-G1-S, but there were no peak in the case of the other OEG-attached PAMAM dendron-lipids. Endothermic peaks for MDEG-DL-G1-S and EDEG-DL-G1-S were detected at 41.5 °C and 40.0 °C, respectively, and these temperatures were different with cloud points. That is, these endothermic peaks in DSC charts are not due to dehydration of the OEG unit attached to PAMAM-DLs, but to the gel-to-liquid crystalline transition of the alkyl chains, indicating that the alkyl chains of MDEG-DL-G1-S and EDEG-DL-G1-S took typical molecular packing in lipid bilayer. In the following experiments combined with PEG-Chol, EDEG-DL-G1-S was selected considering lipid molecular membrane formation and the adjustment of cloud point to around body temperature.

**Figure 4:**
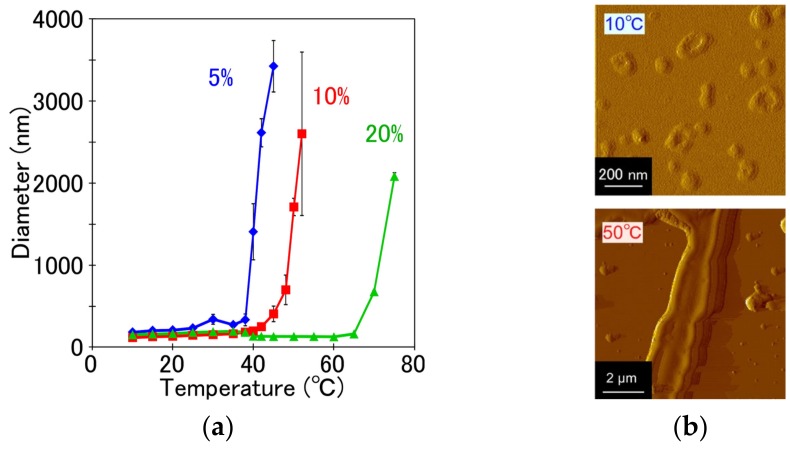
Temperature-dependence of average diameter of EDEG-DL-G1-S/PEG-Chol assemblies with varying compositions (**a**), and AFM images of EDEG-DL-G1-S/PEG-Chol (95/5) assemblies at 10 and 50 °C (**b**). In (a), data are the average of three experiments ± SD.

EDEG-DL-G1-S self-assembly had large aggregates even below the cloud point. For improvement of colloidal stability and the adjustment of cloud point as temperature-responsive nanocarrier, PEG-Chol was combined to EDEG-DL-G1-S self-assembly. Figure 4a shows the change in average diameter of EDEG-DL-G1-S/PEG-Chol assemblies (5%, 10% and 20% PEG-Chol) with an increase in temperature. At low temperature, the colloidal dispersity of EDEG-DL-G1-S assembly was remarkably improved by combining PEG-Chol, and then, EDEG-DL-G1-S/PEG-Chol assemblies with 5%, 10% and 20% PEG-Chol had average diameters with less than 200 nm. Increasing temperature, average diameter of EDEG-DL-G1-S/PEG-Chol assemblies steeply increased, and temperatures exhibiting diameter shifted to higher temperature with an increase in PEG-Chol contents. EDEG-DL-G1-S/PEG-Chol (95/5) assemblies showed suitable change in diameter as temperature-responsive nanocarrier, that is, they exhibited temperature-responsive change of diameter at 39 °C. This temperature-responsive size change was due to morphological change of EDEG-DL-G1-S/PEG-Chol (95/5) assemblies from spherical vesicles to fibrous structures (Figure 4b). Such temperature-responsive morphological change EDEG-DL-G1-S/PEG-Chol (95/5) assemblies was similar with that of IBAM-attached PAMAM-DL assemblies [13]. In the case of IBAM-attached PAMAM-DL assemblies, the hydration of the dendron moiety of the IBAM-attached PAMAM DLs provide molecular packing suitable to take on a lamellar phase. However, the dehydration of the dendron head group induce shrinkage of the dendron head group moiety, which changes its dynamic molecular shape to truncated conic, which is preferable to form an inverted rodlike micelle phase. The morphological change of EDEG-DL-G1-S/PEG-Chol (95/5) assemblies shown in Figure 4 might be induced by same mechanism with IBAM-attached PAMAM-DL assemblies.

**Figure 5:**
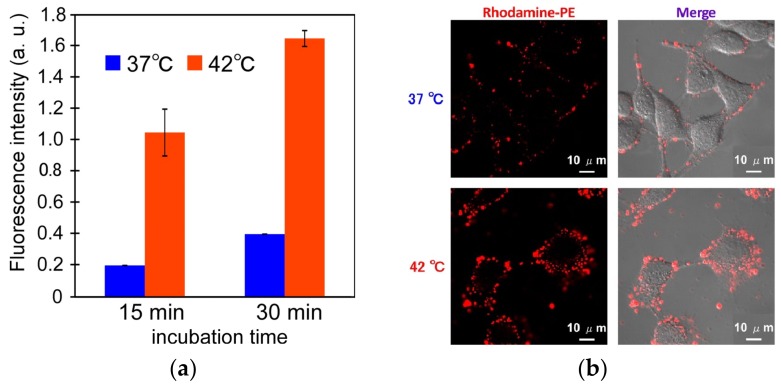
Cellular uptake of EDEG-DL-G1-S/PEG-Chol (95/5) assemblies evaluated by flow cytometry (**a**) and CLSM observation (**b**). In this experiments, EDEG-DL-G1-S/PEG-Chol assemblies were fluorescently labeled using rhodamine−PE. CLSM observation was performed with incubation time of 15 min. In (**a**), data are the average of three experiments ± SD.

**Figure 6:**
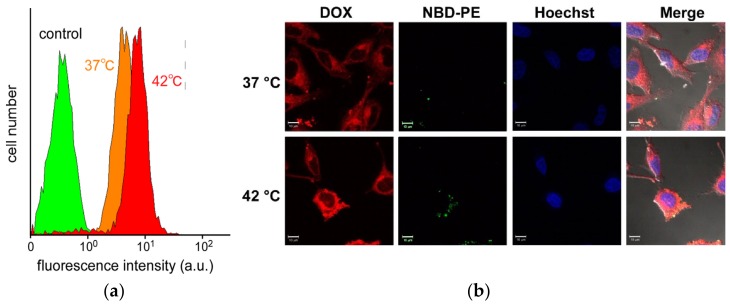
Cellular uptake and intracellular distribution of DOX-loaded EDEG-DL-G1-S/PEG-Chol (95/5) assemblies. (**a**) Cellular uptake evaluated by flow cytometry detected DOX fluorescence; (**b**) CLSM images of HeLa cells treated with DOX-loaded EDEG-DL-G1-S/PEG-Chol assemblies fluorescently labeled using NBD−PE. HeLa cells were incubated with DOX-loaded EDEG-DL-G1-S/PEG-Chol assemblies for 15 min.

Cellular uptake of EDEG-DL-G1-S/PEG-Chol (95/5) assemblies at 37 °C and 42 °C was evaluated by flow cytometry and confocal laser scanning microscopic (CLSM) observation, in which EDEG-DL-G1-S/PEG-Chol assemblies were fluorescently labeled using rhodamine B-sulfonyl phosphatidylethanolamine (rhodamine−PE). Fluorescence intensities of HeLa cells treated with EDEG-DL-G1-S/PEG-Chol (95/5) assemblies increased by both a prolongation of incubation time and an increase in temperature (Figure 5a). When comparing CLSM images at 37 °C and 42 °C (Figure 5b), DOX fluorescence was observed only on the cell surface at 37 °C, whereas at 42 °C., red fluorescence was observed not only intensely but also cellular internalization of assemblies. This might be due to the effect of temperature-responsive morphological change through the dehydration of EDEG moieties, and the hydrophobic interaction might enhance the cellular association of EDEG-DL-G1-S/PEG-Chol assemblies. Cellular uptake and intracellular distribution of DOX-loaded EDEG-DL-G1-S/PEG-Chol assemblies were also evaluated by flow cytometry and CLSM observation (Figure 6). Amount of DOX taken up into the cells at 42 °C were larger than that at 37 °C, and this was agreed with the results on the cellular uptake of the assemblies shown in Figure 5a. The assemblies (NBD-PE) were observed as green dots, whereas DOX red fluorescence were spread in the cytosol. Additionally, in the case of 42 °C, a part of DOX molecules were distributed into nucleus. These results indicated that EDEG-DL-G1-S/PEG-Chol assemblies could deliver DOX molecules into the cells and release DOX molecules from the assemblies.

**Figure 7:**
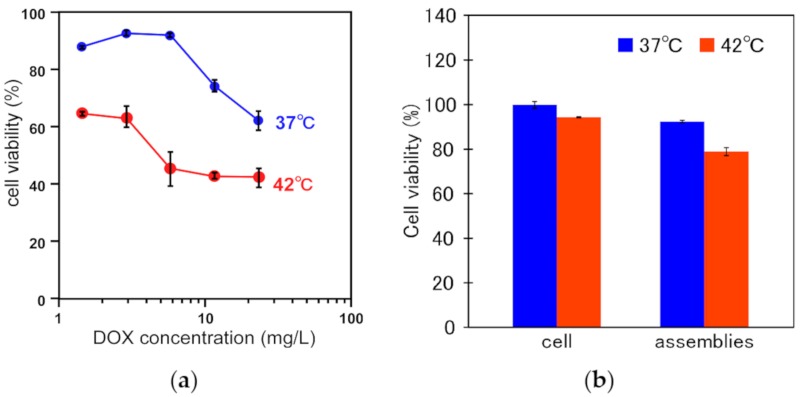
Cell viability of HeLa cells treated with DOX-loaded EDEG-DL-G1-S/PEG-Chol assemblies (**a**) and DEG-DL-G1-S/PEG-Chol assemblies without DOX loading (**b**) at 37 °C and 42 °C. Cell viabilities were evaluated by MTT assay. Data are the average of three experiments ± SD. In (**a**), DOX was loaded into the assemblies at DOX/lipid (mol/mol) = 0.41 ± 0.04. In (**b**), lipid concentration was 0.5 mM, and this concentration corresponds to 5-time higher than 20 mg/L of DOX in DOX-loaded EDGE-DL-G1-S/PEG-Chol assemblies.

Finally, the cell viabilities of HeLa cells treated with DOX-loaded EDEG-DL-G1-S/PEG-Chol assemblies at 37 °C and 42 °C were evaluated by MTT assay (Figure 7a). When the cells were incubated with DOX-loaded assemblies at 37 °C, high cell viabilities were maintained at low DOX concentration and the cell viability decreased at a DOX concentration of 10 mg/L or more. The cell viabilities at 42 °C were decreased compared with those at 37 °C, and this was due to an increased taken-up amount of DOX (Figure 6a) and difference in intracellular distribution of DOX (Figure 6b). Also, EDEG-DL-G1-S/PEG-Chol assemblies without DOX loading were negligible cytotoxicity (Figure 7b). These results indicated the utility of EDEG-DL-G1-S/PEG-Chol assemblies as temperature-responsive nanocarrier.

## 3. Materials and Methods 

### 3.1. Materials

Dioctadecylamine, oleylamine and oleoyl chloride were purchased from Sigma-Aldrich (St. Louis, MO, USA). Methyl acrylate, ethylenediamine, triethylamine (TEA), dimethylsulfoxide (DMSO), *N*,*N*’-dimethylformamide (DMF) and disodium hydrogenphosphate (Na_2_HPO_4_) were purchased from Kishida Chemical (Osaka, Japan). Sodium cyanide, sodium lithium aluminum hydride, chloroform, dichloromethane, acetic anhydride and 3-(4,5-dimethyl-2-thiazoryl)-2,5-diphenyl-2H-tetrazolium bromide (MTT) were purchased from Wako Pure Chemical (Osaka, Japan). SUNBRIGHT CS-010 (Cholesteryl-oxy-poly(ethylene glycol), PEG−Chol; Mw 1000) was kindly provided from NOF Co. (Tokyo, Japan). *N*-(7-nitrobenz-2-oxa-1,3-diazol-4-yl)dioleoyl phosphatidylethanolamine (NBD−PE), rhodamine B-sulfonyl phosphatidylethanolamine (rhodamine−PE) were purchased from Avanti Polar Lipids (Birmingham, AL, USA). Doxorubicin hydrochloride (DOX·HCl) was purchased from Kyowa Hakko Kirin Co., Ltd. (Tokyo, Japan). Hoechst33342 was purchased from Invitrogen (Carlsbad, CA, USA). Merck Kieselgel 60 (230-400 mesh) (Merck KGaA, Darmstadt, Germany) was used for silica gel column chromatography. 4-Nitrophenyl chloroformate-functionalized alkoxy diethylene glycol (EDEG-4-nitrophenyl chloroformate, MDEG-4-nitrophenyl chloroformate) was synthesized as reported [21]. DL−G1−S and DL−G2−S were synthesized according to previous report [22]. Dialysis membrane (Spectra/Por 6, molecular weight cut off: 2000) was purchased from Spectrum laboratories Inc. (Rancho Dominguez, CA, USA). Dulbecco’s modified Eagles’s medium (DMEM) was purchased from Nissui Pharmaceutical (Tokyo Japan). Fetal bovine serum (FBS) was purchased from MP Biomedical Inc. (Santa Ana, CA, USA).

### 3.2. Synthesis of OEG-attached PAMAM-DLs

#### 3.2.1. Synthesis of MDEG−DL−G1−S

To a solution of DL−G1−S (100 mg, 0.12 mmol) in dichloromethane (6 mL), the solution of MDEG-4-nitrophenyl chloroformate (131 mg, 0.48 mmol) in dichloromethane (2 mL) was added dropwise under nitrogen atmosphere. After the reaction for 6 days at room temperature, solvent was evaporated under vacuum, and the residue was chromatographed on silica gel using chloroform−methanol (9/1, 8/2, v/v) as an eluent. The yield was 100 mg (72%). The synthesis was confirmed by ^1^H NMR (Figure S3a).

Calc [M]^+^ (C_63_H_125_N_7_O_11_) m/z, Found ESI-MS [M + H]^+^ m/z 1157.0, [M + Na]^+^ m/z 1178.9.

#### 3.2.2. Synthesis of EDEG−DL−G1−S

DL−G1−S (110 mg, 0.13 mmol) was dissolved in dichloromethane (6 mL), and EDEG-4-nitrophenyl chloroformate (228 mg, 0.76 mmol) in dichloromethane (3 mL) was added dropwise to DL−G1−S solution under nitrogen atmosphere. After the reaction for 7 days at room temperature, solvent was evaporated under vacuum, and the residue was chromatographed on silica gel using chloroform−methanol (9/1, 8/2, v/v) as an eluent. The yield was 100 mg (67%). The synthesis was confirmed by ^1^H NMR (Figure S3b).

Calc [M]^+^ (C_63_H_125_N_7_O_11_) m/z, Found ESI-MS [M + H]^+^ m/z 1185.0, [M + Na]^+^ m/z 1207.0.

#### 3.2.3. Synthesis of MDEG−DL−G2−S

DL−G2−S (110 mg, 83.3 µmol) was dissolved in DMF (6 mL) and MDEG-4-nitrophenyl chloroformate (190 mg, 666.4 µmol) in DMF (2 mL) was added dropwise to DL−G2−S solution under nitrogen atmosphere. After 6 days at room temperature, solvent was evaporated under vacuum, and the residue was chromatographed on silica gel using chloroform−methanol (7/3, 8/2, v/v) as eluent. The yield was 140 mg (88%). The synthesis was confirmed by ^1^H NMR (Figure S3c).

Calc [M]^+^ (C_63_H_125_N_7_O_11_) m/z, Found ESI-MS [M + H]^+^ m/z 1905.4, [M + Na]^+^ m/z 1927.4.

#### 3.2.4. Synthesis of EDEG−DL−G2−S

DL−G2−S (200 mg, 151.5 µmol) was dissolved in dichloromethane (10 mL) and EDEG-4-nitrophenyl chloroformate (544 mg, 1818 µmol) in dichloromethane (3 mL) was added dropwise to DL−G2−S solution under nitrogen atmosphere. After the reaction for 5 days at room temperature, solvent was evaporated under vacuum, and the residue was chromatographed on silica gel using chloroform−methanol (9/1, 8/2, v/v) as eluent. The yield was 120 mg (40%). The synthesis was confirmed by ^1^H NMR (Figure S3d).

#### 3.2.5. Synthesis of MDEG−DL−G1−U

DL−G1−U (400 mg, 0.50 mmol) was dissolved in DMF (6 mL) and MDEG-4-nitrophenyl chloroformate (713 mg, 2.50 mmol) in DMF (3 mL) was added dropwise to DL−G1−U solution under nitrogen atmosphere. After the reaction for 6 days at room temperature, solvent was evaporated under vacuum, and the residue was chromatographed on silica gel using chloroform-methanol (9/1, v/v) as an eluent. The yield was 277 mg (48%). The synthesis was confirmed by ^1^H NMR (Figure S3e).

#### 3.2.6. Synthesis of EDEG−DL−G1−U

DL−G1−U (400 mg, 0.38 mmol) was dissolved in DMF (4 mL), and EDEG-4-nitrophenyl chloroformate (673 mg, 2.25 mmol) in DMF (3 mL) was added dropwise to DL−G1−U solution under nitrogen atmosphere. After the reaction for 7 days at room temperature, solvent was evaporated under vacuum, and the residue was chromatographed on silica gel using chloroform-methanol (9/1, v/v) as an eluent. The yield was 160 mg (37%). Th synthesis was confirmed by ^1^H NMR (Figure S3f).

### 3.3. Preparation of OEG-Attached PAMAM-DLs

A dry thin membrane of the OEG-attached PAMAM-DLs was prepared by solvent evaporation of OEG-attached PAMAM-DLs in CHCl_3_. 10 mM phosphate solution (pH 3.0) was then added to a dry thin membrane and sonicated for 10 min using a bath type sonicator in ice-bath for 60 min to obtain a suspension of lipids.

### 3.4. DOX Loading into OEG-Attached PAMAM-DL Assemblies

DOX·HCl (12 mg, 20.7 µmol) and TEA (6.4 µL, 46.5 µmol) were dissolved in DMSO (6 mL) and stirred overnight under dark. EDEG−DL−G1-S (12 mg, 10.1 µmol) and PEG−Chol (0.55 mg, 0.53 µmol) were dissolved in DMSO (6 mL). DOX solution (6 mL) was mixed with deionized water (6 mL). This mixture was added to the solution dissolving EDEG−DL−G1-S, PEG−Chol and NBD−PE, and the mixture was dialyzed against deionized water using a dialysis membrane bag (molecular weight cut-off of 2000) for 2 days to remove unloaded DOX.

### 3.5. Characterization of OEG-Attached PAMAM-DL Assemblies

Transmittance of the dispersions of OEG-attached PAMAM-DL assemblies in 140 mM NaCl and 10 mM phosphate solution (pH 7.4) were examined. Transmittance of the suspension at 500 nm was measured using a Jasco V-670 spectrophotometer (Jasco Inc., Tokyo, Japan) equipped with a Peltier type thermostatic cell holder coupled with a controller ETC-505T (Jasco Inc., Tokyo, Japan). Before the measurement, the suspension was kept at 10 °C for 30 min in a cell holder.

Differential scanning calorimetric (DSC) measurements were performed with a Nano DSC (TA Instruments Japan Inc). A given amounts of lipid-containing 10 mM phosphate and 140 mM NaCl solution at pH 7.4 was sealed. The heating rate was 1.0 °C min^−1^.

To evaluate the average diameters of OEG-attached PAMAM-DL assemblies, dynamic light scattering (DLS) measurements were performed and analyzed using Zetasizer Nano ZS (Malvern Instruments Ltd, Worcester-shire, UK).

Atomic force microscopic (AFM) observations were performed by SPI3800 probe station and SPA400 unit system of the scanning probe microscopy system (Seiko Instruments Inc, Chiba, Japan). The cantilever was made of silicon (SIDF40; Seiko Instruments Inc, Chiba, Japan), and its spring constant was 38 N/m.

### 3.6. In Vitro Performances of OEG-Attached PAMAM-DL Assemblies

#### 3.6.1. Cellular Uptake

Thin membrane composed of EDEG—DL-G1-S, PEG−Chol and rhodamine−PE (95/5/0.6, mol/mol/mol) was prepared to obtain fluorescent-labeled DL assemblies, and then, fluorescent-labeled EDEG-DL-G1-S/PEG−Chol assemblies were prepared by dispersing PBS. HeLa cells (1.0 × 10^5^ cells) cultured overnight in a 12-well plate were washed with PBS containing 0.36 mM CaCl_2_ and 0.42 mM MgCl_2_ (PBS(+)) and then incubated with culture medium. rhodamine−labeled PAMAM-DL assemblies or DOX-loaded PAMAM-DL assemblies were added to cells incubated for 15 min at 37 °C or 42 °C (5.0 µg DOX/mL). The cells were then rinsed with PBS(+) three times. The detached cells using trypsin were analyzed by a flow cytometer (EPICS XL, Beckman Coulter Inc., Brea, CA, USA).

#### 3.6.2. Intracellular Distribution of DOX-Loaded OEG-Attached PAMAM-DL Assemblies

Thin membrane composed of EDEG−DL-G1-S, PEG−Chol and NBD−PE (95/5/0.6, mol/mol/mol) was used to obtain fluorescent-labeled DOX-loaded DL assemblies. HeLa cells (2 × 10^5^ cells) were seeded into a 35 mm glass-bottom dish in 2 mL of DMEM supplemented with 10% FBS and cultured overnight then washed with PBS(+) and then DMEM without FBS (1.0 mL) was added. DOX-loaded PAMAM-DL assembly suspension was added gently to the cells and incubated for 15 min at 37 °C or 42 °C (23.2 µg DOX/mL). After the incubation, the cells were washed with PBS(+) twice and PBS(−) once, followed by addition of 1 µL of Hoechst33342 (10 mg/mL). After 15 min staining, cells were washed with PBS(+) three times. Confocal laser scanning microscopic (CLSM) analysis was performed using LSM 5 EXCITER (Carl Zeiss Co., Ltd., Oberkochen, Germany).

#### 3.6.3. In Vitro Cytotoxicity Assay

HeLa cells (1.0 × 10^5^ cells) cultured overnight in a 12-well plate were washed with PBS (+) and then incubated with culture medium. The free DOX or DOX-loaded DL assemblies suspension at various concentrations were added to cells incubated for 15 min at 37 °C or 42 °C. Cells were gently washed with PBS twice, and incubated for another 72 h in 1.0 mL of DMEM supplemented with 10% FBS. The culture medium was then replaced with DMEM with 10% FBS containing MTT. 3 h after incubation, the medium was removed, and the cells were solubilized in isopropanol containing 0.1 M HCl. The viable cells were counted from the absorbance at 490 nm using Microplate Reader (SH-8000 CORONA ELECTRIC Co., Ltd., Ibaraki, Japan).

## 4. Conclusions

Several types of OEG-attached PAMAM-DLs were synthesized by changing the generation of PAMAM dendron, saturated and unsaturated chains as alkyl chains, and terminal OEGs for the purpose of the development of temperature-responsive nanocarrier. These OEG-attached PAMAM-DLs dispersions showed cloud points depending on their compositions. EDEG-DL-G1-S/PEG-Chol assemblies could deliver DOX molecules into HeLa cells, and an incubation temperatures, i.e. below and above cloud point, influenced to the cellular uptake of DOX-loaded EDEG-DL-G1-S/PEG-Chol assemblies and intracellular distribution of the delivered DOX molecules. In vitro anticancer effect (cell viability) of DOX-loaded EDEG-DL-G1-S/PEG-Chol assemblies was promoted by inducing DOX release with temperature-responsive morphological change. The results obtained here should provide valuable information that will aid in the design of temperature-responsive nanocarrier using lipid molecules.

## Figures and Tables

**Table 1:** Cloud points of OEG-attached PAMAM-DLs dispersions.

Code	Terminal Moiety	Dendron Generation	Alkyl Chains	Cloud Point ^1^
MDEG-DL-G2-S	MDEG EDEG MDEG EDEG MDEG EDEG	2nd 2nd 1st 1st 1st 1st	saturated saturated saturated saturated unsaturated unsaturated	68 °C 34 °C 34 °C 23 °C 45°C 26 °C
EDEG-DL-G2-S MDEG-DL-G1-S EDEG-DL-G1-S MDEG-DL-G1-U EDEG-DL-G1-U				

^1^ These temperatures were determined from Figure 2.

## Supplementary Materials

The following are available online at https://www.mdpi.com/2079-4983/11/1/16/s1, Figure S1: Change in average diameters of OEG-attached PAMAM-DLs dispersions with increase in temperature, Figure S2: Temperature-dependence of transmittance of EDEG-DL-G2-S at varying pHs and the relationship between cloud points and pHs for EDEG-DL-G2-S, Figure S3. ^1^H NMR spectra of the synthesized OEG-attached PAMAM DLs. (CDCl_3_, 400 MHz). (a) MDEG-DL-G1-S; (b) EDEG-DL-G1-S; (c) MDEG-DL-G2-S; (d) EDEG-DL-G2-S; (e) MDEG-DL-G1-U; (f) EDEG-DL-G1-U.
