# Supplementary material for: Temperature-Responsive Molecular Assemblies Using Oligo(Ethylene Glycol)-Attached Polyamidoamine Dendron Lipids and their Functions as Drug Carriers
Source: J Funct Biomater. 2020 Mar 13;11(1):16. doi: 10.3390/jfb11010016 (PMC7151555; doi:10.3390/jfb11010016)
Supplement: Supplementary file 1 [file jfb-11-00016-s001.pdf]

Journal of Functional Biomaterials

Article

Temperature-Responsive Molecular Assemblies Using Oligo(Ethylene Glycol)-Attached Polyamidoamine Dendron Lipids and their Functions as Drug Carriers

Takuya Hashimoto, Yuji Hirai, Eiji Yuba *, Atsushi Harada * and Kenji Kono

Department of Applied Chemistry, Graduate School of Engineering, Osaka Prefecture University, 1-1 Gakuen-cho, Naka-ku, Sakai, Osaka 599-8531, Japan; sv108049@edu.osakafu-u.ac.jp (T.H.); 35615u@ube-ind.co.jp (Y.H.); biopolymer@chem.osakafu-u.ac.jp (K.K.)

* Correspondence: harada@chem.osakafu-u.ac.jp (A.H.); yuba@chem.osakafu-u.ac.jp (E.Y.)

Received: 10 February 2020; Accepted: 11 March 2020; Published: 13 March 2020

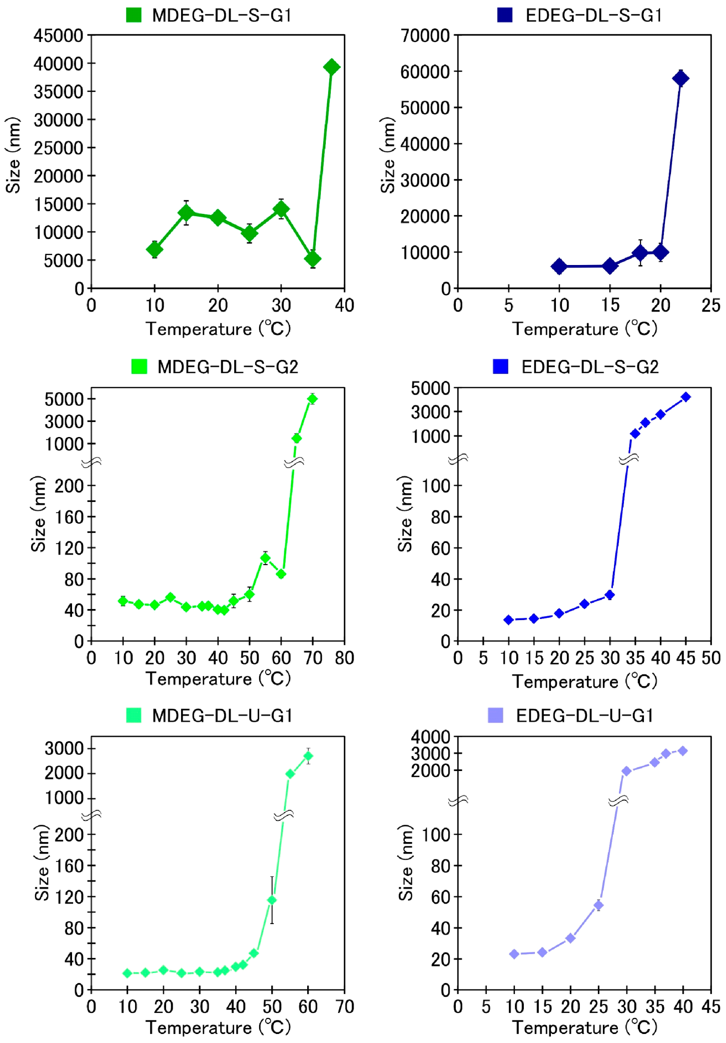

Figure S1. Change in average diameters of OEG-attached PAMAM-DLs dispersions with increase in temperature. OEG-attached PAMAM-DLs were dispersed in in 10 mM phosphate and 140 mM NaCl at pH 7.4.

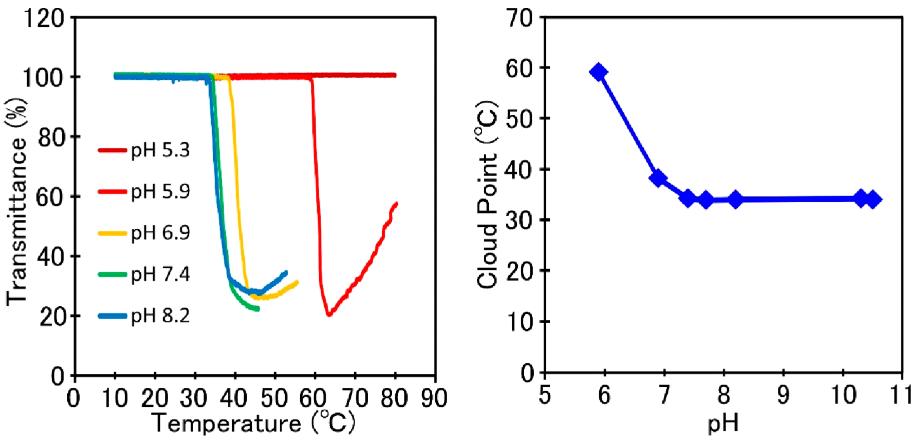

(**a**) (**b**)

Figure S2. Temperature-dependence of transmittance of EDEG-DL-G2-S at varying pHs (**a**) and the relationship between cloud points and pHs for EDEG-DL-G2-S (**b**).

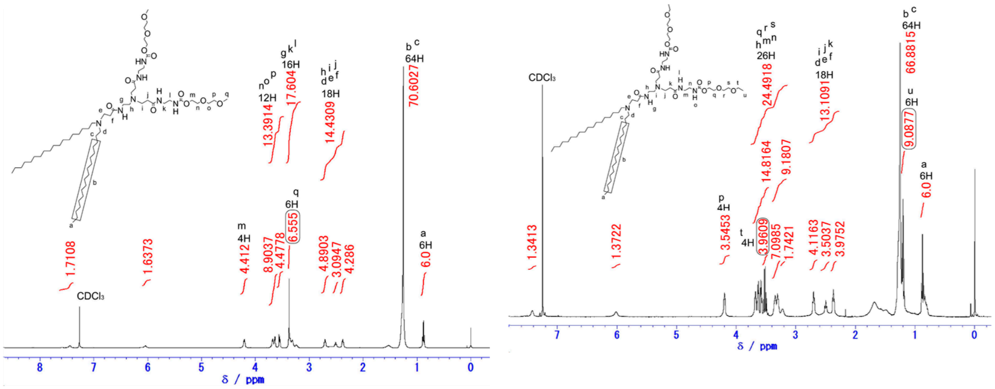

(**a**) (**b**)

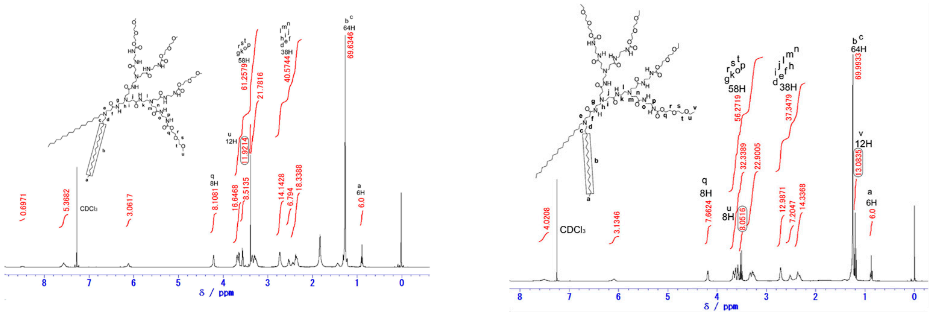

(**c**) (**d**)

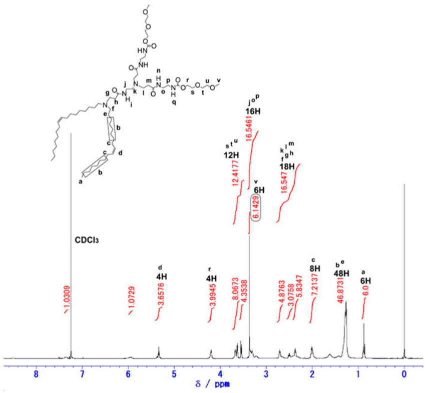

(**e**)

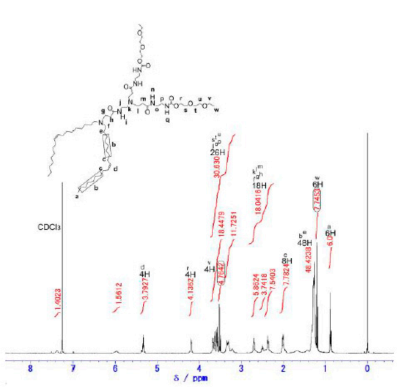

(**f**)

Figure S3. ^{1}H NMR spectra of the synthesized OEG-attached PAMAM DLs. ($CDCl_3$, 400 MHz). (**a**) MDEG-DL-G1-S; (**b**) EDEG-DL-G1-S; (**c**) MDEG-DL-G2-S; (**d**) EDEG-DL-G2-S; (**e**) MDEG-DL-G1-U; (**f**) EDEG-DL-G1-U.
